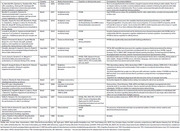# Narrative review: Cognitive evaluation for vehicle driving in South America

**DOI:** 10.1002/alz70857_107019

**Published:** 2025-12-26

**Authors:** Valeria Contreras, Bettina Aguiar

**Affiliations:** ^1^ UdelaR, Montevideo, Montevideo, Uruguay

## Abstract

**Background:**

An increase in life expectancy implies an increase in people with cognitive impairment and dementia who request a driver's license. While it is accepted that subjects with moderate or severe dementia are disqualified from driving, this does not happen in mild stages. People with cognitive impairment have an increased risk suffering traffic accidents. In Uruguay, as in many South American countries, the cognitive evaluation for the driving license is not protocolized. An investigation is being carried out to identify which neuropsychological tests predict better driving performance.

**Objective:**

To find South American publications regarding cognitive evaluation in people with cognitive impairment and older adults.

**Method:**

A comprehensive search was carried out in PubMed, LILACS and SciELO for articles from countries in South America with the terms “vehicular driving”, “cognitive impairment”, “dementia”, “older adults” and synonyms. The search was extended to Google Scholar, adding as a filter that the terms were in the title.

**Results:**

19 articles were obtained. Five were eliminated because they weren’t related to the objective of the analysis. Of the remaining 14, we highlight a clinical practice guideline and three neuropsychological batteries, all from Argentina. The guideline is based on a systematic review of literature. We also found 4 other revisions with international information.

We found 7 cross‐sectional studies that performed cognitive assessments, very dissimilar to each other. All used the Mini Mental State Examination (MMSE). In five MMSE was found useful, while in another it was reported as inconsistent, which was also described in a review.

Other tests described as correlated with driving were: Digit Symbols Test, Boston Naming Test (BNT), Trail Making Tests, Semantic Verbal Fluency, AD8 Interview, Logical Memory, Verbal and Visual Learning Test, Frontal Assessment Battery, Neuropsychiatric Inventory, and Functional Assessment Questionnaire.

The conclusions of the studies are summarized in Table 1.

**Conclusion:**

There is little evidence in South America regarding the best form of cognitive assessment for driving in older adults or those with cognitive impairment. The available information highlights the relevance of the assessment of executive functions and BNT, as well as the importance of obtaining information from multiple sources.